# Lung cancers attributable to environmental tobacco smoke and air pollution in non-smokers in different European countries: a prospective study

**DOI:** 10.1186/1476-069X-6-7

**Published:** 2007-02-15

**Authors:** Paolo Vineis, Gerard Hoek, Michal Krzyzanowski, Federica Vigna-Taglianti, Fabrizio Veglia, Luisa Airoldi, Kim Overvad, Ole Raaschou-Nielsen, Francoise Clavel-Chapelon, Jacob Linseisen, Heiner Boeing, Antonia Trichopoulou, Domenico Palli, Vittorio Krogh, Rosario Tumino, Salvatore Panico, H Bas Bueno-De-Mesquita, Petra H Peeters, Eiliv Lund E, Antonio Agudo, Carmen Martinez, Miren Dorronsoro, Aurelio Barricarte, Lluis Cirera, J Ramon Quiros, Goran Berglund, Jonas Manjer, Bertil Forsberg, Nicholas E Day, Tim J Key, Rudolf Kaaks, Rodolfo Saracci, Elio Riboli

**Affiliations:** 1 Imperial College London, London UK (Paolo Vineis: and University of Torino, Italy); 2 Department of Environmental and Occupational Health, Utrecht University, Utrecht, The Netherlands; 3 World Health Organization, European Centre for Environment and Health, Bonn, Germany; 4 ISI Foundation, Turin, Italy; 5 Istituto di Ricerche Farmacologiche Mario Negri, Milan, Italy; 6 Department of Clinical Epidemiology, Aalborg Hospital, Aarhus University Hospital, Aalborg, Denmark; 7 Institute of Cancer Epidemiology, Danish Cancer Society, Copenhagen, Denmark; 8 INSERM, (Institut National de la Santé et de la Recherche Médicale), ERI 20, EA 4045, and Institut Gustave Roussy, Villejuif, F-94805, France; 9 Division of Clinical Epidemiology, Deutsches Krebsforschungszentrum, Heidelberg, Germany; 10 German Institute of Human Nutrition, PotsdamRehbücke, Germany; 11 Department of Hygiene and Epidemiology, Medical School, University of Athens, Greece; 12 Molecular and Nutritional Epidemiology Unit, and Molecular Biology Laboratory, CSPO-Scientific Institute of Tuscany, Florence, Italy; 13 Department of Epidemiology, National Cancer Istitute, Milan, Italy; 14 Cancer Registry, Azienda Ospedaliera "Civile M.P. Arezzo", Ragusa, Italy; 15 Dipartimento di Medicina Clinica e Sperimentale, Università Federico II, Naples, Italy; 16 Centre for Nutrition and Health, National Institute for Public Health and the Environment, Bilthoven, The Netherlands; 17 Julius Center for Health Sciences and Primary Care, University Medical Center, Utrecht, The Netherlands; 18 Institute of Community Medicine, University of Tromso, Norway; 19 Department of Epidemiology, Catalan Institute of Oncology Barcelona, Consejería de Sanidad y Servicios Sociales, Spain; 20 Andalusian School of Public Health, Granada, Spain; 21 Department of Public Health of Guipuzkoa, San Sebastian, Spain; 22 Public Health Institute, Navarra, Spain; 23 Department of Epidemiology, Regional Health Council, Murcia, Spain; 24 Public Health and Health Planning Directorate, Asturias, Spain; 25 Malmö Diet and Cancer Study, Lund University, Malmö, Sweden; 26 Dept of Surgery, Malmö University Hospital, Malmö, Sweden; 27 Department of Public Health and Clinical Medicine, University of Umeå, Sweden; 28 MRC Dunn Human Nutrition Unit, Cambridge, UK; 29 Cancer Research UK Epidemiology Unit, University of Oxford, UK; 30 International Agency for Research on Cancer, Lyon, France; 31 "IFC-National Research Council, Pisa, Italy"; 32 Department of Epidemiology and Public Health, Imperial College London, St Mary's Campus, Norfolk Place, London W2 1PG, UK

## Abstract

**Background:**

Several countries are discussing new legislation on the ban of smoking in public places, and on the acceptable levels of traffic-related air pollutants. It is therefore useful to estimate the burden of disease associated with indoor and outdoor air pollution.

**Methods:**

We have estimated exposure to Environmental Tobacco Smoke (ETS) and to air pollution in never smokers and ex-smokers in a large prospective study in 10 European countries (European Prospective Investigation into Cancer and Nutrition)(N = 520,000). We report estimates of the proportion of lung cancers attributable to ETS and air pollution in this population.

**Results:**

The proportion of lung cancers in never- and ex-smokers attributable to ETS was estimated as between 16 and 24%, mainly due to the contribution of work-related exposure. We have also estimated that 5–7% of lung cancers in European never smokers and ex-smokers are attributable to high levels of air pollution, as expressed by NO_2 _or proximity to heavy traffic roads. NO_2 _is the expression of a mixture of combustion (traffic-related) particles and gases, and is also related to power plants and waste incinerator emissions.

**Discussion:**

We have estimated risks of lung cancer attributable to ETS and traffic-related air pollution in a large prospective study in Europe. Information bias can be ruled out due to the prospective design, and we have thoroughly controlled for potential confounders, including restriction to never smokers and long-term ex-smokers. Concerning traffic-related air pollution, the thresholds for indicators of exposure we have used are rather strict, i.e. they correspond to the high levels of exposure that characterize mainly Southern European countries (levels of NO_2 _in Denmark and Sweden are closer to 10–20 ug/m^3^, whereas levels in Italy are around 30 or 40, or higher).

Therefore, further reduction in exposure levels below 30 ug/m^3 ^would correspond to additional lung cancer cases prevented, and our estimate of 5–7% is likely to be an underestimate. Overall, our prospective study draws attention to the need for strict legislation concerning the quality of air in Europe.

## Background

From the point of view of public health it is important to estimate the burden of disease related to different air pollutants, in view of the new legislation that several countries are now introducing. Environmental tobacco smoke (ETS) and traffic-related air pollution share a few characteristics: they are widespread exposures in both the developed and the developing countries; they have several chemical components in common, in particular Polycyclic Aromatic Hydrocarbons; and they have been associated with increased risks of lung cancer and other diseases. The relative lung cancer increase is approximately of the same entity for both (around 20–30%) [1-4]. However, no single prospective study has tried to assess the effects of both exposures in the same populations with accurate and standardized methods, and to estimate the total burden of lung cancer attributable to the two exposures.

We have estimated exposure to ETS and to air pollution in the context of the large EPIC study (European Prospective Investigation into Cancer and Nutrition), and here we report estimates of the attributable risks at the population level.

## Methods

EPIC (European Prospective Investigation into Cancer and Nutrition) [5] is a multi-center European study, in which more than 520,000 healthy volunteers have been recruited in 10 European countries (Sweden, Denmark, Norway, Netherlands, UK, France, Germany, Spain, Italy, Greece) [6]. The cohort includes subjects of both genders, in the age range 35–74 at recruitment. Recruitment took place between 1993 and 1998. Dietary information on the frequency of consumption of more than 120 foods and drinks has been obtained by a Food Frequency Questionnaire, validated in a pilot phase. At enrollment, weight, height, waist and hip circumferences have been measured for each participant. Detailed information has been collected on reproductive history, physical activity, smoking and alcohol drinking history, medical history, occupation, education level and other socioeconomic variables; the questionnaire was printed in two separate versions for men and women. A computerized central database has been developed after checking, coding and quality control procedures.

The lifestyle questionnaire included several questions on smoking habits. Present exposure to environmental tobacco smoke (ETS) was investigated by a few questions: (a) exposure to ETS yes/no; (b) place of exposure (home, work); (c) number of hours of current exposure; (d) number of cigarettes smoked by the spouse in the presence of the index subject; (e) exposure to ETS during childhood. Only half of the EPIC centers (11/22) (in France, Italy, Denmark, Sweden, The Netherlands and Potsdam, Germany) included questions on ETS in the questionnaire, and most of these collected information only on items (a) or (b) above.

The EPIC cohort has been followed-up since inception through Cancer Registries, vital statistics (mortality), and – in some areas – hospital discharge data. Whenever available, diagnosis is based on histological confirmation. All incident cancers and all causes of death are registered and checked centrally.

We have also nested a case-control study on lung cancer (newly diagnosed after recruitment) within the EPIC cohort, aiming at studying the relationship with air pollution through individual exposure assessment (which was not feasible for the whole cohort). We included only never smokers or ex-smokers who quit at least 10 years before recruitment. We have matched three controls per case. Matching criteria were gender, age (plus or minus 5 years), smoking status, country of recruitment, and time elapsed between date of recruitment and date of diagnosis. Matching was introduced to allow strict control of potentially confounding variables, considering that other risk factors may be stronger than ETS or air pollution. The study has been approved by the Ethical Committee of the International Agency for Research on Cancer, and by all the local Ethical Committees of the participating centers.

Exposure to air pollution was assessed using concentration data from monitoring stations in routine air quality monitoring networks. We excluded traffic and industrial network sites and instead focused on urban or rural background locations, i.e. the site should be at least 50 meters away from any major road and at least 100 meters from a freeway and not located in an industrial area (preferably in a residential area). Data were obtained through searching AIRBASE, the air pollution database from the European Topic Center on Air Quality in Bilthoven [7]. In addition we contacted national/local monitoring agencies using a questionnaire and used Internet sites from national agencies. The average concentration for O_3_, SO_2_, NO_2_, CO and PM10 from all background monitoring stations in the city of residence was assigned to each study subject. For each home address we also assessed whether the home was located in a major street (yes/no). Several studies have documented substantial differences in concentration of traffic-related pollutants between traffic and background locations. For all homes, we used detailed Internet maps to evaluate whether the home was located in a major street (yes/no). Details are given in reference [8].

For ETS we have analyzed the whole cohort by Cox's proportional hazards model, using age as the dependent variable as suggested by Korn et al [9]. Hazard ratios (HR) were adjusted by gender, smoking habits (former or never smoker), time since recruitment, country, school years, energy intake, fruit and vegetables consumption, and physical activity. In the nested case-control study on air pollution we have computed odds ratios (OR) and 95% confidence intervals (CI) in conditional logistic regression models [10] that included educational level (4 categories), as a further adjustment variable in addition to matching variables. We repeated the analyses using the center of recruitment and smoking duration/amount as additional adjustment variables. Analyses were performed with the SAS package (Cary, NC, USA) for a personal computer. Population attributable risks percent (PAR) were computed as:

G(OR-1)/(G(OR-1)+1)

where G is the proportion of exposed controls [11].

Details on main results, including cotinine measurements and information on genetic susceptibility, have been published previously [6,8]. Here we focus on the public health impact of both exposure to ETS and air pollution.

## Results

Table 1 shows the prevalence of self-reported exposure to ETS in EPIC (whole cohort). An estimate between 40% and 60% can be reasonably considered as representative of most European countries, with the notable exception of Germany, with a much lower prevalence. These figures are comparable with those from other previous surveys [4,12].

**Table 1:** Distribution of exposure to Environmental Tobacco Smoke in the EPIC cohort ^1^

	**No**	**Yes**	**% exposed**
France	23821	35433	59.8
Italy	6953	13817	66.5
The Netherlands	7849	13474	63.2
Germany	19279	5592	22.5
Sweden	6673	15455	69.8
Denmark	10216	45786	81.8

^1: ^according to country (exposure at home and/or at work)

Table 2 shows hazard ratios for ETS and odds ratios for air pollution indicators. Concerning air pollution, we have found a statistically significant association only with exposure to NO_2 _(OR = 1.30, 95% CI 1.02–1.66). Estimates of the OR were 1.05 (95% CI 0.65–1.69; greater or equal to 27 vs. lower than 27 ug/m^3^) for PM10, and 1.15 (0.92–1.43; greater or equal to 11 vs. lower than 11 ug/m^3^) for SO_2_. The OR for NO_2 _was also adjusted for potential confounding from ETS by including cotinine levels in the logistic regression models. The cotinine-adjusted OR was 1.62 (95% CI 0.92–1.43). Among never smokers the association with NO_2 _was represented by an OR of 1.09 (CI 0.78–1.52), while in ex-smokers the OR was 1.59 (1.10–2.30). Concerning ETS, the estimates were 1.05 for never smokers (0.60–1.82) and 2.32 for ex-smokers (0.94–5.71); after adjustment for smoking duration and number of cigarettes smoked the relative risk remained unchanged – for details see [6]. We found little heterogeneity among study centers, with a p-value for interaction with country of 0.94 for ETS and 0.76 for NO_2_. However, the sample size did not allow the estimation of risks for individual centers. The odds ratio for the joint exposure to both ETS and NO_2 _was as high as 4.51, but the CI was very wide (0.46–43.48).

**Table 2:** Hazard Ratios (HR) (whole cohort) and Odds Ratios (OR) (nested case-control study)^1^

**ETS**	**Hazard ratios**	**Controls:**
		**exposed/total**

Exposure to ETS		
at home and/or at work	1.34 (0.85 to 2.13)	71722/123479 (58%)
PAR%	0.16	
		
ETS at work	1.65 (1.04 to 2.63)	58653/123479 (47%)
PAR%	0.24	
		
ETS at home	1.03 (0.60 to 1.76)	23396/123479 (19%)
PAR%	0.006	
		
ETS in childhood, daily (a)	2.00 (0.94 to 4.28)	10282/60182 (17%)
PAR%	0.14	
		
Air pollution	Odds ratios	
Living near heavy		
traffic road	1.46 (0.89–2.40)	242/1990 (12%)
PAR%	0.05	
		
Exposure to NO2 (b)	1.30 (1.02–1.66)	428/1562 (27%)
PAR%	0.07	

(a) Reference category: never or seldom exposed to ETS in childhood; never smokers only

(b) more than 30 microgr/m^3 ^vs. less than 30

^1^: 95% confidence intervals (in parenthesis) for ETS exposure (home and/or work), exposure to indicators of air pollution and lung cancer. Both OR and HR are adjusted by gender, age (plus or minus 5 years), smoking (former or never smoker), country, school years, energy intake, fruit and vegetable consumption, and physical activity. PAR% = population attributable risk % (see text).

In previous publications we have shown more detailed analyses stratified by different characteristics of the cohort, but here we focus only on the main associations to estimate population attributable risks. On this basis we estimated for ETS an attributable proportion of 16% based on the analysis of the whole cohort. In fact, as shown before [6], the risk is mainly concentrated in work-related exposures, with a higher attributable risk (around 24%). For air pollution, for which only the case-control data are available, the estimate is approximately 5–7% with both indicators used (heavy traffic road and NO_2 _greater than 30 ug/m^3^).

## Discussion

A previous investigation estimated that approximately 7 per thousand cases of lung cancer could be attributed to ETS exposure in never smokers (spouses of smokers) [13]. Our figure is much larger, but it is based on both ex-smokers and never smokers. If we consider never smokers, the Hazard Risk estimate is 1.05 (95% CI 0.60–1.82) and the attributable risk in the population is 2.5%, still higher than the mentioned previous estimate. A likely explanation is that the above-mentioned study considered only exposure at home, whereas in our study the most important source of exposure, associated with higher risks, was exposure at work. The attributable risk for home exposure in our study, in fact, is 6 per thousand, in line with the previous estimate. In another recent study among never smokers only [14], the estimated OR was 1.31 (1.03–1.67) with a very high proportion of subjects who were ever exposed to ETS (86%). This gives an attributable proportion as high as 21%. When only exposure at home was considered, the OR was 1.19 (1.01–1.40), the proportion exposed 48% and the PAR% 8%; for workplace exposure, OR = 1.11 (0.94–1.31), proportion exposed 39%, PAR% 4%.

Particularly concerning is the association between childhood exposure and the risk of lung cancer in adulthood (Table 2). As we have previously reported, there was a statistically significant trend (p = 0.018) with increasing time of exposure to ETS in childhood, and with a hazard ratio of 3.63 (95% CI 1.19–11.1211) for daily exposure for many hours [6]. To our knowledge, ours is the first prospective study to report such association [15,16]. Of course, the reliability of information on exposure to ETS in childhood can be questioned, although most people are expected to recall whether their parents smoked. The uptake of carcinogens by children exposed to ETS is widespread and quantitatively important [17]. Fetuses and newborns seem to be particularly susceptible to carcinogens. In one study, mother-newborn pairs exposed to high levels of indoor pollution from coal smoke were investigated [18]. For all markers, including DNA adducts, newborns had levels which were higher than in the mothers, although tranplacental exposure levels were 10-times lower than the paired mother exposures. In a series of well-designed experiments, Somers et al [19] reported increased mutation rates in herring gulls and mice exposed to air pollution at levels that characterize normal urban environments.

Our estimate of 5–7% attributable to air pollution (measured as proximity to heavy traffic roads or exposure to NO_2_) is consistent with previous estimates from other authors [20]. However, both different exposure metrics and different pollutants have been used for previous estimates. In particular, Kunzli et al [21] used PM10, while estimates from the American Cancer Society (ACS) study [22] used PM2.5 as the reference pollutant. The ACS based the attributable risk calculation on a linear increase in PM2.5 in the 7.5–50 ug/m^3 ^range. Unfortunately we do not have PM2.5 data in Genair, due to the sparse availability of such exposure parameter in Europe. Therefore, our estimate is compatible but not strictly comparable to the ACS estimate. Also, we did not find a clear association with PM10 in our data. SO_2 _as well (an indicator of long-term exposure to stationary combustion) was not associated in a statistically significant manner with lung cancer in our study, consistently with two Scandinavian lung cancer investigations [23,24]. A caveat is needed, i.e. we should consider that our cohort was never meant to be representative for the general population. This may be important especially for the estimates of the prevalence of relevant exposures. The PARs that we have computed apply to EPIC participants, but should be extrapolated with caution to lung cancer in non- and past smokers in the European population at large. However, the prevalence estimates we have computed in our study are, at least for ETS, realistic (with the exception of Germany) and similar to figures from other surveys. Another limitation of our study-related to the restriction to non-smokers – is the small number of subjects with the ensuing large confidence intervals of estimates.

We have excluded current smokers and recent ex-smokers. An interaction has been previously described between recent smoking and exposure to air pollution [25], but we were not able to address it; nor were we able to estimate the attributable risk due to the joint effect of the two exposures. This is clearly a limitation of our study, which, however, has the advantage of avoiding confounding through restriction to never and ex-smokers.

In summary, in our large prospective study we have found that among never smokers and ex-smokers since at least 10 years lung cancer was associated to self-reported ETS exposure at the time of recruitment. The proportion of lung cancers in never- and ex-smokers attributable to ETS was estimated as between 16 and 24%, mainly due to the contribution of work-related exposure. Also exposure to ETS in childhood seemed to be associated with a relatively high proportion of lung cancers in adulthood; although this observation needs to be confirmed, it is particularly relevant for its public health implications. We have also found that higher exposure to some air pollutants (in particular NO_2_) can increase the risk of lung cancer in non-smokers. We have thoroughly controlled for potential confounders. Information bias can be ruled out due to the prospective design. Changes in exposure levels are likely to have occurred after the cohort recruitment; this and other measurement errors are likely to lead to a blurring of the differences between cases and non-cases, i.e. to an underestimation of the strength of associations. The association with NO_2 _does not necessarily imply that this single pollutant is (more) carcinogenic than others, but could express the lower degree of exposure misclassification with NO_2_. The hypothesis that NO2 could better represent, at least in Europe, exposure to other pollutants, e.g. fine or ultrafine particles, has been extensively discussed, in particular in the recent revision of the WHO World Air Quality Guidelines [26]. We have estimated that 5–7% of lung cancers in European never smokers and ex-smokers are attributable to high levels of air pollution, as expressed by NO_2 _or proximity to heavy traffic roads. The latter indicator has limitations, mainly related to the fact that it is associated to social class. It should be noticed that the thresholds for indicators of air pollution exposure we have used are rather strict, i.e. they correspond to the high levels of exposure that characterize mainly Southern European countries (levels of NO_2 _in Denmark and Sweden are closer to 10–20 ug/m^3^, whereas levels in Italy are around 30 or 40, or higher). Therefore, further reduction in exposure levels below 30 ug/m^3 ^would correspond to additional lung cancer cases prevented, and our estimate of 5–7% is likely to be an underestimate. Overall, our prospective study draws attention to the need for strict legislation concerning the quality of air in Europe.

## Abbreviations

▪ OR: odds ratios

▪ CI: confidence intervals

▪ PAR: population attributable risk

▪ ETS: environmental tobacco smoke

## Competing interests

The author(s) declare that they have no competing interests.

## Authors' contributions

All authors have read and approved the final version of this paper. All authors have directly participated in the planning, execution or analysis of the study:

PV, ER and RS carried out conception and design;

KO, O R-N, FC, HB, VK, DP, SP, RT, B B-De-M, PP GB, GH and PV carried out acquisition of data;

LA, FV, LO, RP, HA, AD, SG, EG, PH, CM, GM and MP carried out laboratory analyses, data analysis and interpretation of data;

PV drafted the article. He takes responsibility for the integrity of the data and the accuracy of the data analysis (guarantor).

## References

[B1] Hackshaw AK, Law MR, Wald NJ (1997). The accumulated evidence on lung cancer and environmental tobacco smoke. Bmj.

[B2] Vineis P, Alavanja M, Buffler P, Fontham E, Franceschi S, Gao YT, Gupta PC, Hackshaw A, Matos E, Samet J, Sitas F, Smith J, Stayner L, Straif K, Thun MJ, Wichmann HE, Wu AH, Zaridze D, Peto R, Doll R (2004). Tobacco and cancer: recent epidemiological evidence. J Natl Cancer Inst.

[B3] IARC (2004). Tobacco smoke and involuntary smoking. IARC Monogr Eval Carcinog Risks Hum.

[B4] Benowitz NL (1999). Biomarkers of environmental tobacco smoke exposure. Environ Health Perspect.

[B5] Riboli E, Hunt KJ, Slimani N, Ferrari P, Norat T, Fahey M, Charrondiere UR, Hemon B, Casagrande C, Vignat J, Overvad K, Tjonneland A, Clavel-Chapelon F, Thiebaut A, Wahrendorf J, Boeing H, Trichopoulos D, Trichopoulou A, Vineis P, Palli D, Bueno-De-Mesquita HB, Peeters PH, Lund E, Engeset D, Gonzalez CA, Barricarte A, Berglund G, Hallmans G, Day NE, Key TJ, Kaaks R, Saracci R (2002). European Prospective Investigation into Cancer and Nutrition (EPIC): study populations and data collection. Public Health Nutr.

[B6] Vineis P, Airoldi L, Veglia P, Olgiati L, Pastorelli R, Autrup H, Dunning A, Garte S, Gormally E, Hainaut P, Malaveille C, Matullo G, Peluso M, Overvad K, Tjonneland A, Clavel-Chapelon F, Boeing H, Krogh V, Palli D, Panico S, Tumino R, Bueno-De-Mesquita B, Peeters P, Berglund G, Hallmans G, Saracci R, Riboli E (2005). Environmental tobacco smoke and risk of respiratory cancer and chronic obstructive pulmonary disease in former smokers and never smokers in the EPIC prospective study. Bmj.

[B7] EEA EEA AirBase - the European Air quality dataBase.

[B8] Vineis P, Hoek G, Krzyzanowski M, Vigna-Taglianti F, Veglia F, Airoldi L, Autrup H, Dunning A, Garte S, Hainaut P, Malaveille C, Matullo G, Overvad K, Raaschou-Nielsen O, Clavel-Chapelon F, Linseisen J, Boeing H, Trichopoulou A, Palli D, Peluso M, Krogh V, Tumino R, Panico S, Bueno-De-Mesquita HB, Peeters PH, Lund EE, Gonzalez CA, Martinez C, Dorronsoro M, Barricarte A, Cirera L, Quiros JR, Berglund G, Forsberg B, Day NE, Key TJ, Saracci R, Kaaks R, Riboli E (2006). Air pollution and risk of lung cancer in a prospective study in Europe. Int J Cancer.

[B9] Korn EL, Graubard BI, Midthune D (1997). Time-to-event analysis of longitudinal follow-up of a survey: choice of the time-scale. Am J Epidemiol.

[B10] Breslow NE, Day NE (1980). Statistical methods in cancer research. Volume I - The analysis of case-control studies. IARC Sci Publ.

[B11] Rothman KJ GS (1998). Modern epidemiology.

[B12] Veglia F, Vineis P, Berrino F, Cerulli Ddel S, Giurdanella MC, Tumino R, Fiorini L, Sacerdote C, Panico S, Mattiello A, Palli D, Saieva C, Davico L (2003). Determinants of exposure to environmental tobacco smoke in 21,588 Italian non-smokers. Tumori.

[B13] Tredaniel J, Boffetta P, Saracci R, Hirsch A (1997). Non-smoker lung cancer deaths attributable to exposure to spouse's environmental tobacco smoke. Int J Epidemiol.

[B14] Brennan P, Buffler PA, Reynolds P, Wu AH, Wichmann HE, Agudo A, Pershagen G, Jockel KH, Benhamou S, Greenberg RS, Merletti F, Winck C, Fontham ET, Kreuzer M, Darby SC, Forastiere F, Simonato L, Boffetta P (2004). Secondhand smoke exposure in adulthood and risk of lung cancer among never smokers: a pooled analysis of two large studies. Int J Cancer.

[B15] Lee CH, Ko YC, Goggins W, Huang JJ, Huang MS, Kao EL, Wang HZ (2000). Lifetime environmental exposure to tobacco smoke and primary lung cancer of non-smoking Taiwanese women. Int J Epidemiol.

[B16] Tredaniel J, Boffetta P, Little J, Saracci R, Hirsch A (1994). Exposure to passive smoking during pregnancy and childhood, and cancer risk: the epidemiological evidence. Paediatr Perinat Epidemiol.

[B17] Hecht SS, Ye M, Carmella SG, Fredrickson A, Adgate JL, Greaves IA, Church TR, Ryan AD, Mongin SJ, Sexton K (2001). Metabolites of a tobacco-specific lung carcinogen in the urine of elementary school-aged children. Cancer Epidemiol Biomarkers Prev.

[B18] Whyatt RM, Jedrychowski W, Hemminki K, Santella RM, Tsai WY, Yang K, Perera FP (2001). Biomarkers of polycyclic aromatic hydrocarbon-DNA damage and cigarette smoke exposures in paired maternal and newborn blood samples as a measure of differential susceptibility. Cancer Epidemiol Biomarkers Prev.

[B19] Somers CM, Yauk CL, White PA, Parfett CL, Quinn JS (2002). Air pollution induces heritable DNA mutations. Proc Natl Acad Sci U S A.

[B20] Cohen AJ, Ross Anderson H, Ostro B, Pandey KD, Krzyzanowski M, Kunzli N, Gutschmidt K, Pope A, Romieu I, Samet JM, Smith K (2005). The global burden of disease due to outdoor air pollution. J Toxicol Environ Health A.

[B21] Kunzli N, Kaiser R, Medina S, Studnicka M, Chanel O, Filliger P, Herry M, Horak F, Puybonnieux-Texier V, Quenel P, Schneider J, Seethaler R, Vergnaud JC, Sommer H (2000). Public-health impact of outdoor and traffic-related air pollution: a European assessment. Lancet.

[B22] Pope CA, Burnett RT, Thun MJ, Calle EE, Krewski D, Ito K, Thurston GD (2002). Lung cancer, cardiopulmonary mortality, and long-term exposure to fine particulate air pollution. Jama.

[B23] Nyberg F, Gustavsson P, Jarup L, Bellander T, Berglind N, Jakobsson R, Pershagen G (2000). Urban air pollution and lung cancer in Stockholm. Epidemiology.

[B24] Nafstad P, Haheim LL, Oftedal B, Gram F, Holme I, Hjermann I, Leren P (2003). Lung cancer and air pollution: a 27 year follow up of 16 209 Norwegian men. Thorax.

[B25] Holgate S SJ, Samet JM CAJ (1999). Air Pollution and Health. Air Pollution and Lung Cancer.

[B26] WHO (2006). Air quality guidelines for particulate matter, ozone, nitrogen dioxide and sulfur dioxide - Global update 2005 - Summary of risk assessment.

